# Immune Checkpoint Inhibitor Endocrinopathies—Insights From 3 Unusual Cases

**DOI:** 10.1210/jcemcr/luaf238

**Published:** 2025-10-24

**Authors:** Eystein S Husebye

**Affiliations:** Department of Clinical Science, University of Bergen, Bergen N-5021, Norway; Department of Medicine, Haukeland University Hospital, Bergen N-5021, Norway

**Keywords:** immune checkpoint inhibitor, primary adrenal insufficiency, thyroiditis, hypophysitis, type 1 diabetes, autoimmune lipodystrophy

Immune checkpoint inhibitors (ICIs) are monoclonal antibody drugs that have revolutionized cancer therapy and led to dramatically improved survival for aggressive cancers such as malignant melanoma and lung cancer. However, these benefits come with the cost of significant immune-related side effects. Endocrine glands are among those commonly affected, giving rise to potentially fatal complications if not promptly diagnosed and treated [1].

ICI-induced endocrinopathies affect up to 15% of patients treated [2] depending on the drug, dose, and duration of treatment. In general, the combination of cytotoxic T-lymphocyte associated protein 4 (CTLA-4) and programmed cell death protein 1 (PD-1) inhibitors have a more toxic effect than either drug class used alone. Thyroiditis is the most common endocrine complication, followed by hypophysitis, whereas other glands are rarely targeted [2]. Symptoms are often unspecific and overlap with those from the malignant disease and oncological treatments, making diagnosis and treatment challenging. Three cases in *JCEM Case Reports* [3-5] highlight many of the features of these endocrinopathies and provide important learning points.

Rossi et al [3] describe a 39-year-old male who developed polyendocrinopathy while being treated with the PD-1-inhibitor pembrolizumab. Only 3 weeks after receiving his first dose, he developed thyrotoxicosis and started methimazole treatment. However, because he rapidly reverted to hypothyroidism, methimazole was discontinued and levothyroxine replacement initiated.

This course is typical for ICI-mediated destructive thyroiditis that often has a transient thyrotoxic phase followed by hypothyroidism. Graves’ disease is rarely seen in association with ICI treatment, and methimazole should not be used without clear evidence of hyperthyroidism and presence of thyrotropin (TSH) receptor antibodies [1]. Treatment with β blockers is usually sufficient to control thyrotoxic symptoms and many require life-long levothyroxine to treat the subsequent hypothyroidism, although some recover [6].

The 39-year-old male continued pembrolizumab for another 3 months but was then admitted acutely with fever, dizziness, asthenia, headache, and hyponatremia, indicating an adrenal crisis. ACTH and cortisol levels were low, and he was started on glucocorticoid replacement. The other pituitary axes were normal, although TSH was elevated (11.7 IU/L; reference range, 0.35-3.5) indicating underreplacement with levothyroxine.

Hypophysitis is the second most common endocrinopathy associated with ICI treatment affecting approximately 6%, especially when CTLA-4 inhibitors are used alone or in combination with PD-1 antagonists [2]. Hypophysitis associated with PD1 inhibitors in monotherapy are milder, often with isolated ACTH deficiency as in this case. Sudden and unexpected development of hyponatremia should prompt testing of adrenal function and start of glucocorticoid replacement without delay. In severe cases, the pituitary increases in size causing severe headache and visual problems by compressing the optic chiasm, but often there are no local symptoms, and the pituitary appears normal on imaging. Unfortunately, ACTH deficiency is almost always permanent and requires lifelong replacement therapy, but the other pituitary axes can normalize. High-dose steroid treatment is not recommended except in cases with large pituitary glands causing visual problems [1]. Pharmacological steroid doses given over time can also cause adrenal insufficiency.

So far, Rossi et al's case was rather typical for ICI-induced endocrinopathies. However, a year later, when he was evaluated for hypogonadism, ACTH was elevated (320.9 pg/mL [SI: 70.6 pmol/L]) (reference range, 8-60 pg/mL [SI: 1.8-13.2 pmol/L]) and cortisol low (0.21 µg/dL [SI: 5.7 nmol/L]) (reference range, 5-23 µg/dL [SI: 137.9-634.4 nmol/L) compatible with primary adrenal insufficiency, also supported by high renin, low aldosterone, and low dehydroepiandrosterone sulphate levels. With this unexpected switch from secondary to primary adrenal insufficiency the patient also needed replacement therapy with a mineralocorticoid. Only a few cases of ICI-induced primary adrenal insufficiency have been reported [7], many displaying autoantibodies against 21-hydroxylase, the biomarker for autoimmune primary adrenal insufficiency, but this was not found in Rossi et al's case.

Another serious complication of ICI treatment is type 1 diabetes. Tang et al report a 72-year-old female with a metastatic gastrointestinal stromal tumor who was diagnosed with type 2 diabetes 4 months after starting treatment with pembrolizumab [4]. She was discharged with metformin and continued pembrolizumab. The following 3 years, she had several episodes with diabetic ketoacidosis (DKA) but also received treatment with a sodium glucose cotransporter 2 inhibitor during this time, a drug class known to precipitate DKA. Finally, she was tested for autoimmune diabetes, and high levels of autoantibodies against glutamic acid decarboxylase were found, as was a very low C-peptide level. Insulin treatment was started.

ICI-induced type 1 diabetes typically has an unusually acute onset, often with DKA and glycosylated hemoglobin levels only modestly elevated. Many carry the human leukocyte antigen genotypes associated with regular type 1 diabetes and display the autoantibodies typically seen in type 1 diabetes, including autoantibodies against glutamic acid decarboxylase [8]. Although pembrolizumab might have contributed to her autoimmune diabetes, sodium glucose cotransporter-2 inhibitor treatment could also explain the DKA episodes.

Another autoimmune complication to ICI treatment, acquired lipodystrophy, can also lead to diabetes mellitus resulting from severe insulin resistance. The third case by Kennedy et al reported a 48-year-old female with metastatic melanoma who, on combination therapy with ipilimumab and nivolumab, developed severe lipodystrophy, panniculitis, and diabetes mellitus [5]. This very rare complication to ICI treatment is associated with autoantibodies against perilipin-1, the key enzyme mobilizing triglycerides from adipose tissue.

Taken together, ICI-induced endocrinopathies are common and caused by immune activation. Because symptoms and findings can be unspecific, screening tests are recommended. The European Society of Endocrinology guidelines recommend a test battery including TSH, free thyroxine, cortisol, glucose, and electrolytes (sodium, potassium, calcium) in a morning sample at baseline and every 4 to 6 weeks, preferably before each treatment cycle. Testing of pituitary hormones including a morning ACTH, luteinization hormone, FSH, estradiol (premenopausal females), testosterone (males), and prolactin is recommended when there an increased risk of hypophysitis, especially when CTLA-4 and PD-1 inhibitors are combined. Finally, it is also important to inform the patients about side effects and how to act if symptoms appear.

## Disclosures

No conflict of interest related to this manuscript.

## Data Availability

Original data generated and analyzed for this case report are included in this published article.
